# Effect of Water Extract of Mangosteen Pericarp on Donepezil Pharmacokinetics in Mice

**DOI:** 10.3390/molecules26175246

**Published:** 2021-08-30

**Authors:** Mingoo Bae, Seung Yon Han, Eun-Sun Kim, Byung Hoon You, Young-Mi Kim, Jungsook Cho, Young-Won Chin, Young Hee Choi

**Affiliations:** 1 College of Pharmacy and Integrated Research Institute for Drug Development, Dongguk University_Seoul, 32 Dongguk-lo, Ilsandong-gu, Goyang-si 10326, Gyonggi-do, Korea; nophra88@naver.com (M.B.); hsyglory@gmail.com (S.Y.H.); ikhhycs@naver.com (E.-S.K.); hoon4131@nate.com (B.H.Y.); neuroph@dongguk.edu (J.C.); 2 Research Institute of Pharmaceutical Sciences, College of Pharmacy, Seoul National University, 1, Gwanak-ro, Gwanak-gu, Seoul 08826, Korea; 0210121@hanmail.net (Y.-M.K.); ywchin@snu.ac.kr (Y.-W.C.)

**Keywords:** donepezil, water extract of mangosteen pericarp, pharmacokinetic interaction, tissue distribution, brain

## Abstract

The pharmacokinetic (PK) change in a drug by co-administered herbal products can alter the efficacy and toxicity. In the circumstances that herb–drug combinations have been increasingly attempted to alleviate Alzheimer’s disease (AD), the PK evaluation of herb–drug interaction (HDI) is necessary. The change in systemic exposure as well as target tissue distribution of the drug have been issued in HDIs. Recently, the memory-enhancing effects of water extract of mangosteen pericarp (WMP) has been reported, suggesting a potential for the combination of WMP and donepezil (DNP) for AD treatment. Thus, it was evaluated how WMP affects the PK change of donepezil, including systemic exposure and tissue distribution in mice after simultaneous oral administration of DNP with WMP. Firstly, co-treatment of WMP and donepezil showed a stronger inhibitory effect (by 23.0%) on the neurotoxicity induced by Aβ_(25–35)_ in SH-SY5Y neuroblastoma cells than donepezil alone, suggesting that the combination of WMP and donepezil may be more effective in moderating neurotoxicity than donepezil alone. In PK interaction, WMP increased donepezil concentration in the brain at 4 h (by 63.6%) after administration without affecting systemic exposure of donepezil. Taken together, our results suggest that WMP might be used in combination with DNP as a therapy for AD.

## 1. Introduction

Alzheimer’s disease (AD) is the most common form neurodegenerative disease accompanying progressive memory and cognitive impairment [1,2,3]. The accumulation of senile plaques composed by the aggregated β-amyloid (Aβ), formation of neurofibrillary tangle as aggregates of phosphorylated tau protein, and progressive loss of cholinergic neurons and nicotinic acetylcholine receptors have been characterized as main pathological features of AD [4,5,6]. Until now, acetylcholinesterase inhibitors [7,8,9,10] and N-methyl-D-aspartate (NMDA) receptor antagonists [7,11] have been used to alleviate the cognitive symptoms associated with AD. Donepezil hydrochloride (Aricept^®^), a reversible acetylcholinesterase inhibitor, is popularly used because it has less hepatic toxicity [7,8,9]. Apart from the acetylcholinesterase inhibitory activity, other pharmacological properties of donepezil for neuroprotection have been suggested, such as the rescue effects on Aβ-induced apoptosis and hippocampal long-term potentiation impairment [12,13,14], and the promoting effect on oligodendrocyte differentiation in Aβ-induced neuroral cell death [12].

The safety and antioxidant activity of the polar fraction of mangosteen (*Garcinia mangostana* L.) have already been identified in humans [15,16], and a number of clinical evaluations have proved its efficacies [16,17,18]. The aqueous layer of mangosteen extract was effective against lead-induced acetylcholinesterase dysfunction, cognitive impairment [19], and scopolamine-induced memory impairment in mice [20]. Xanthones, as active components of mangosteen extracts, have shown efficacies against neurodegenerative diseases such as Alzheimer’s disease, Parkinson’s disease, and amyotrophic lateral sclerosis [21]. Interestingly, the pericarp of mangosteen contains at least 50 different bioactive compounds, such as xanthones, polyphenols, and catechins [18,22,23]. These extracts show diverse biological activities, including antioxidant, anti-inflammatory, and neuroprotective activities [21,24,25]. Recently, our colleagues demonstrated that water extract of mangosteen pericarp (WMP) possesses a memory-enhancing effect through antioxidative neuroprotection and anti-apoptotic action [26], providing support for the clinical use of WMP in single and combination applications.

Due to the complex and multifactorial etiologies of AD, various approaches have been attempted to explore the favorable therapeutic outcomes in AD [1,27,28,29,30]. Numerous extracts of herbal products containing diverse and active compounds have gained attention as candidates for AD treatment [31,32,33,34,35,36], and therapeutic approaches concerning multi-target drugs (or compounds) for AD treatment have been attempted [2,31]. Furthermore, herb–drug combination therapies have been attempted as a multi-target strategy with successful cases for mitigating symptoms of AD [1,32,33,34]. However, the unexpected efficacy and toxicity have also sometimes occurred [36]. Regulatory agencies (e.g., FDA, EMA, etc.) suggest that the evaluation of the pharmacokinetic herb–drug interactions [37,38,39,40], as well as of PK-based HDIs (e.g., herbal product combinated with donepezil as a drug for AD treatment), is also necessary [3,41].

Until now, the effect of WMP on the pharmacological activity and pharmacokinetic profile of donepezil in combination with WMP has not been investigated. Thus, we firstly evaluated the combinational effect of donepezil with WMP against Aβ-induced neurotoxicity in SH-SY5Y neuroblastoma cells. More importantly, we explored the effect of WMP on donepezil pharmacokinetics in mice, especially focusing on the donepezil concentration change in systemic exposure and the brain as a pharmacological target tissue of AD to provide an underlying mechanism to lead to in vivo efficacy or toxicity in donepezil and WMP combination.

## 2. Results

### 2.1. Effect of WMP and Donepezil Hydrochloride (DNP) Combination on Cell Viability

**Figure 1:**
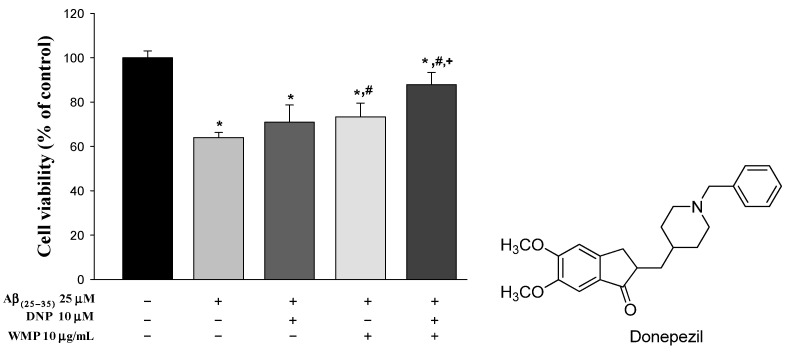
Effect of WMP and DNP combination on Aβ_(25–35)_-induced neurotoxicity in SH-SY5Y neuroblastoma cells. Each value represents the mean ± S.E.M. from at least three independent experiments. * *p* < 0.05 v.s. vehicle-treated control group; # *p* < 0.05, vs. Aβ_(25–35)_ group; and + *p* < 0.05, DNPWMP was significantly different from other groups, respectively.

The combinational effect of WMP and DNP on cell viability of SH-SY5Y neuroblastoma cells was evaluated using MTT assay. Aβ_(25–35)_ treatment significantly reduced the cell viability (by 36.0%) compared to vehicle treatment. Treatment of WMP or DNP plus WMP (DNPWMP) significantly alleviated the neurotoxicity induced by Aβ_(25–35)_ (by 19.9% and 36.4% in WMP and DNPWMP treatments, respectively) compared to Aβ_(25–35)_ treatment. In addition, DNPWMP treatment significantly increased the cell viability against Aβ_(25–35)_-induced neurotoxicity compared to DNP and WMP treatment (by 23.0 and 13.7%, respectively). These results indicated that DNPWMP combination enhanced the cell viability in SH-SY5Y neuroblastoma cells-induced neurotoxicity by Aβ_(25–35)_ compared to single treatment of DNP or WMP (Figure 1).

### 2.2. Safety of WMP

All mice were alive after oral administration of WMP at doses of 20–500 mg/kg for seven days, and the result showed that the oral LD_50_ of WMP was more than the 500 mg/kg by Behrens approach [42]. There was no significance of food and water consumption in all groups of mice: the food intakes were 2.73 ± 0.512, 2.64 ± 0.351, 2.71 ± 0.452, and 2.88 ± 0.315 g/day/mouse for 20, 50, 100 and 500 mg/kg administration, respectively, and the water consumptions were 3.95 ± 0.248, 4.12 ± 0.525, 3.99 ± 0.348, and 4.21 ± 0.449 mL/day/mouse, respectively. Any behavioral change was not observed in WMP administration compared to vehicle administration.

### 2.3. Effect of WMP on the Pharmacokinetics of Donepezil

**Figure 2:**
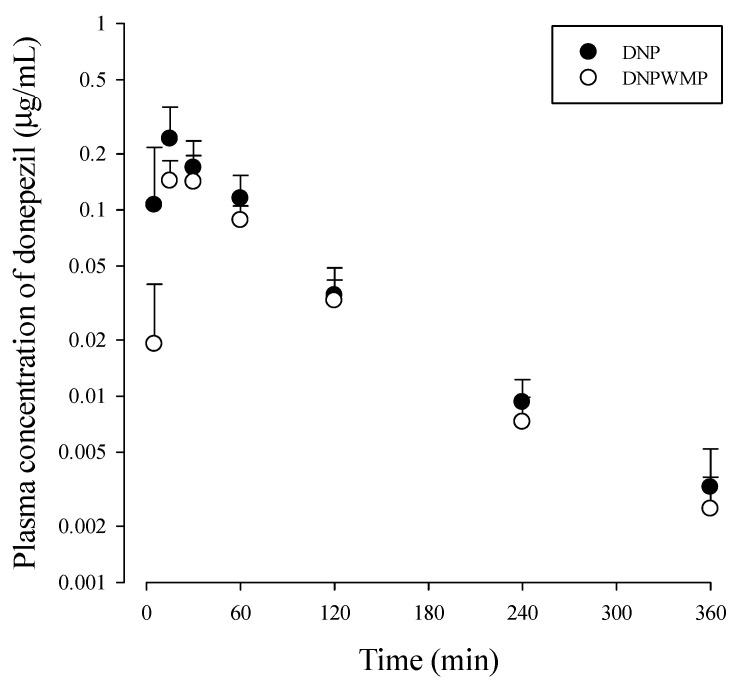
Plasma concentration-time curve of donepezil after simultaneously oral administration of DNP (as 10 mg/kg of donepezil; *n* = 10 from 30 mice) with and without WMP (300 mg/kg; *n* = 10 from 28 mice) to mice. Three or four blood samples were obtained from each mouse by cardiac puncture, which produced ten sets of PK data. Each value represents the mean ± S.D.

The mean arterial plasma concentration‒time profiles of donepezil in DNP and DNPWMP groups are shown in Figure 2, and relevant pharmacokinetic parameters are listed in Table 1. There was no significant difference in all PK parameters between the two groups.

### 2.4. Effect of WMP on Tissue Distribution of Donepezil

Concentrations and the ratios of tissue-to-plasma concentrations (T/P ratios) of donepezil at 1 and 4 h in various tissues were listed in Table 2. In both groups, concentrations of donepezil in plasma and tissues rapidly decreased as the time passed. Donepezil concentrations in all tissues were higher than plasma concentration at the same time. In the brain, as a pharmacological target tissue of donepezil, donepezil concentration in the brain was higher than plasma, although donepezil concentration in the brain was relatively lower than other tissues in both groups. Interestingly, donepezil concentration and its T/P ratio in the brain at 4 h were significantly higher (by 63.6% and 55.5%, respectively) than DNP alone. Except for the significantly lower donepezil concentration in the large intestine of the DNPWMP compared to the DNP group, there was no change of donepezil concentrations in other tissues between two groups at 4 h. At 1 h, there was no difference in donepezil concentrations and its T/P ratios in all tissues between two groups.

Moreover, there was no difference in the identification of the tentative metabolites of donepezil in both groups of plasma and liver samples. Although the quantitative determination of donepezil metabolites could not be conducted, the patterns of the possible metabolites of donepezil were similar.

Based on the main MS/MS fragments of donepezil, the spectra of donepezil consisted of *m*/*z* 380.3, 362.2, 288.3, 273.7, 243.4, 205, 192, 189, 172, 151, and 91.2, with a retention time (RT) of 13.2 min. The parent ions of the tentative metabolites of donepezil showed *m*/*z* 542 at 9.9 min (M1), 366 at 11.6 min (M2), 366 at 11.9 min (M3), 396 at 11.3 min (M4), 396 at 14.0 min (M5), 382 at 10.8 min (M6), and 382 at 11.2 min (M7), respectively. The parent fragment ion signal of M1, *m*/*z* 542, was formed by O-demethylation/glucuronidation, because the fragment ions of *m*/*z* 542, 366, 274, 229, and 91.1 were formed observed. The M2 and M3 might be formed by O-demethylation, in which the fragment ions *m*/*z* 366, 348, 274, 259, 229, 175, 137, and 91.2 were observed. In the case of M4 and M5, phenyl hydroxylation (at 11.3 min) and N-oxidation (at 14 min) were proposed, because the fragment ions of M4, *m*/*z* 396, 378, 286, 241, 204, and 91.2, and those of M5, *m*/*z* 396, 288, 273, 243, 189, and 91.2, were observed. The parent fragment ion signals of M6 and M7, *m*/*z* 382, were formed by O-demethylation/hydroxylation, because the fragment ions of *m*/*z* 382, 364, 290, 272, and 91.3 at 10.8 min and *m*/*z* 382, 364, 272, and 91.3 at 11.2 min were observed. In addition, the parent ion of donepezil, *m*/*z* 380, and its daughter ion fragment, *m*/*z* 91.2, were observed as the same pattern of the tentative metabolites of donepezil. Based on these fragment spectrums, O-methylation/glucuronidation, O-methylation, phenyl hydroxylation, N-oxidation, and O-methylation/hydroxylation were proposed as metabolic pathways of donepezil. These proposed metabolites of donepezil were similar to the previous report [43]. Their spectrums were presented in supplementary data.

### 2.5. Effect of WMP on Plasma Protein Binding of Donepezil in Mice

When a rapid equilibrium dialysis (RED) device (molecular weight cutoff of 8.0 KDa; Thermo scientific, Waltham, MA, USA) was incubated for 4 h at 37 °C and 250 rpm, the plasma protein bindings of donepezil (1 µg/mL) with and without WMP (1 µg/mL) were 80.8 ± 4.22 and 79.8 ± 0.354%, respectively. There was no significant difference between the two groups. For validation, the plasma protein binding value of 1 µg/mL metformin as a low protein binding drug was 9.43%, similar to the reported value [44].

### 2.6. Effect of WMP on Donepezil Metabolism In Vitro S9 Fractions of Liver and Small Intestine

The disappeared percentages of donepezil were 54.7 ± 12.8 and 64.2 ± 3.59% in the liver S9 fractions of the DNP and DNPWMP groups, respectively, and those values were comparable. The corresponding values were 14.9 ± 4.73 and 19.0 ± 7.06% in small intestine S9 fractions of the DNP and DNPWMP groups, respectively. These values were also comparable between two groups. Moreover, the tentative metabolites were observed using precursor and/or product ion scan modes, and the possible metabolites of donepezil by O-methylation/glucuronidation, O-methylation, phenyl hydroxylation, N-oxidation, and O-methylation/hydroxylation were similarly suggested in both groups.

## 3. Discussion

The intensive attempts to develop disease-modifying therapeutics targeting Aβ or tau protein, neuroprotective strategies, and immunotherapies for AD treatment have proceeded; however, there has been no new approved drug for the treatment of AD since 2003 [38]. The situation supports that a combination therapies based on drugs and/or active compounds improving the pathologies associated with AD are valuable. Although the main purpose of this study was to evaluate the effect of WMP on donepezil pharmacokinetics in DNP and WMP combination, we firstly provided the enhanced cell viability of donepezil with WMP against Aβ-induced neurotoxicity in SH-SY5Y neuroblastoma cells compared to single treatment of DNP or WMP which was observed (Figure 1). As an underlying mechanism of donepezil to inhibit Aβ-induced neurotoxicity in SH-SY5Y neuroblastoma cells, other pharmacological properties such as activation of PI3K/Akt and/or Sigma-1 receptor have been proposed: donepezil protected Aβ-induced cytotoxicity through promoting oligodendrocyte differentiation by activating the PI3K/Akt pathway [12,45]. Also, the activation of the Sigma-1 receptor by donepezil antagonized the suppresive action of Aβ on long-term potentiation in rat hippocampi [14,46]. Donepezil has been found to bind to the Sigma-1 receptor with high affinity, regulating a variaty of cellular functions such as Ca^2+^ signaling, neurotransmitter release, cellular protection against Aβ-induced neurotoxicity, and others [14]. As an underlying mechanim of WMP, the inhibition of caspase 3 activation, DNA fragmentation, and ROS suppression of WMP against Aβ-induced neurotoxicity [19,26] can be suggested. Although the pharmacological activity of herbal extract is not a sum of pharmacological activity of individual compounds included in the herbal extract, the efficacy of α- and γ-mangostin, as main active constituents of WMP, for Alzheimer’s disease treatment can somewhat be related to the neuroprotective effect of WMP [16,47,48]. For example, α-mangostin was clarified to treat AD disease through anti-cholinesterase, anti-amyloid-cascade, anti-inflammation and anti-oxidative activities [47], and improved memory impairment by blocking the TAK1/NF-κB signaling pathway [49]. γ-Mangostin was also known to have a neuroprotective activity through inhibiting H_2_O_2_-induced DNA fragmentation and glutamate-induced mitogen-activated protein kinase phosphorylation [48]. Based on these mechanisms of DNP and WMP, DNPWMP treatment showed the enhancement of the protective activity of DNP against Aβ-induced neurotoxicity in SH-SY5Y neuroblastoma cells.

Considering a potential for the use of DNP and WMP together suggested by Figure 1, the effect of WMP on donepezil pharmacokinetics was evaluated. The doses of 10 mg/kg donepezil and 300 mg/kg WMP, respectively, were chosen considering the effective dose of each compound against scopolamine-induced spatial memory impairment in mice reported by our colleagues [26]. After oral administration of DNP with and without WMP in mice, the detection of donepezil in plasma from the early blood sampling time points (5, 15, or 30 min) and the early *C*_max_ [i.e., short time to reach *C*_max_ (*T*_max_)] at 15‒30 min were observed in both groups, indicating the rapid gastrointestinal absorption of donepezil from the GI. Also, the comparable *T*_max_ of donepezil between the two groups meant that the absorption rate of donepezil was not changed by WMP. The percentage of unabsorbed and/or biliary excreted donepezil at 24 h after its oral administration (*GI*_24h_) was the sum of the unabsorbed and biliary excreted fraction of donepezil, and the *GI*_24h_ values of 0.0361 and 0.0798% of the oral dose in the DNP and DNPWMP groups indicated the extensive absorption and negligible biliary excretion of donepezil in both groups. This result was confirmed, as the PK profile of donepezil in Table 1 was similar to the previous report [43]: Matsui et al. [43] reported that donepezil was absorbed rapidly and completely, i.e., 30 min of *T*_max_ and approximately 86.8% of systemic bioavailability, and the biliary excretion ratio of donepezil was 0.16% of its oral dose when [^14^C] donepezil was orally administered in rats. Thus, it could be concluded that WMP did not affect the rapid and complete absorption as well as biliary excretion pathway of donepezil. In humans, the slow absorption of donepezil in the gut, reaching peak concentration within 3–4 h, was reported [50,51,52,53], which could be due to the different formation of donepezil such as orally disintegrating tablets or an oral film-coated tablet affecting its oral absorption [50,51].

In the elimination of donepezil, renal excretion seemed to be minor, considering 1.65 and 0.73% of the percentage of renally excreted donepezil into urine for 24 h (*Ae*_0–24h_) in DNP and DNPWMP groups, respectively. This suggested that, while some donepezil is excreted into the urine in mice, the metabolism of donepezil may be the main route of its elimination. Similarly, Matsui et al. [43] reported that hepatic metabolism of donepezil is a main pathway of elimination. 36.9% of [^14^C]donepezil was excreted into urine, including 1.52% of donepezil and the remaining percentage, 35.4%, of donepezil’s metabolites, in rats after oral administration of [^14^C]donepezil. As its main metabolite excreted into urine, O-glucuronide of donepezil was suggested [43]. In this study, O-methylation/glucuronidation, O-methylation, phenyl hydroxylation, N-oxidation, and O-methylation/hydroxylation were proposed as metabolic pathways of liver (Supplementary data 1). Those metabolites were detected in in vivo liver samples in a tissue distribution study and an in vitro metabolism study using S9 fraction of liver. However, the intensities of the fragment ions of these tentative metabolites were lower in S9 fraction of small intestines in the in vitro metabolism study (in our unpublished data), supporting that the hepatic metabolism of donepezil might be extensive compared to that in the small intestine.

In in vitro donepezil metabolism studies using S9 fraction of liver, the extensive metabolism of donepezil was observed: 54.7% of donepezil was disappeared. This means that donepezil is mainly metabolized in liver as a main elimination route of donepezil, which was similar in the case of co-existence of WMP (64.2%). In other words, WMP did not alter the renal excretion and extensive hepatic metabolism of donepezil. In systemic exposure of donepezil, no change in AUC and *C*_max_ of donepezil indicated that WMP did not affect the systemic exposure of donepezil due to the comparable absorption and elimination pathways of donepezil between the two groups (Figure 1 and Table 1).

In the tissue distribution, the T/P ratios of donepezil above 1 in all tissues indicated that donepezil was distributed well to tissues. Tissue distribution patterns of donepezil were almost unchanged by WMP co-treatment (except liver at 1 h and brain and large intestine at 4 h), suggesting that the effect of WMP on donepezil distribution into tissues might be negligible except in the brain. The increased donepezil concentration in the liver at 1 h by WMP co-treatment (Table 2) may have a negligible effect on the PK property of donepezil, because the AUC (Table 1) and in vitro metabolism of donepezil with and without WMP were comparable. Moreover, the *GI*_24h_ values between DNP and DNPWMP groups meant that the reduced donepezil concentration in the large intestine at 4 h was little contribution to the *GI*_24h_ of donepezil. Interestingly, WMP increased donepezil concentration and T/P ratio in the brain at 4 h (by 63.6% and 55.5%, respectively), indicating that WMP may have the effect of enhancing the pharmacological activity of donepezil in the brain as a pharmacological target tissue. Considering that donepezil concentration in the brain at 1 h was not changed by WMP, it may take some time for WMP to affect donepezil distribution to the brain (Table 2). Especially, approximately 20~30-fold of higher donepezil concentration in the brain than its plasma concentration reported by Matsui et al. [43] strengthened the reproducibility of our result in Table 2. Also, Matsui et al. [43] reported that the ratio of donepezil to [^14^C]donepezil in the brain was 86.9 to 93% in rats, indicating that the permeability of donepezil’s metabolites through the blood-brain barrier was low.

Numerous delivery systems of donepezil targeting into the brain have been developed, resulting in the improvement of donepezil efficacy as a reversible cholinesterase inhibitor used for the treatment of AD [54,55,56,57]. In addition, tissue distribution can provide information necessary to explain the mechanism of action considering the pharmacological target organ, and the increased concentration of a drug in the brain by co-treated compounds have been suggested as a potential therapy for the treatment of AD [35,58,59].

Although further studies on how WMP affects donepezil distribution in the brain are necessary, the following possible mechanisms can be suggested based on the literature. As the first possible mechanism, it can be suggested that WMP may increase donepezil concentration in the brain probably due to the inhibiting P-gp in the blood-brain barriers (BBB). Spieler et al. [60] reported that donepezil concentration in the brain and the cerebrum to plasma ratio of donepezil were increased in P-glycoprotein (P-gp) deficient mice compared to control, indicating that the brain bioavailability of donepezil depends on P-gp. Considering that numerous reports the inhibitory effects of mangosteen pericarp extract and xanthones as main components of mangosteen pericarp extract against P-gp [61,62], the increased donepezil concentration in the brain may be due to the inhibition of P-gp-mediated donepezil efflux through BBB by WMP. Although P-gp is expressed in many other organs, the degree of P-gp inhibition by P-gp inhibitors are different because the concentrations of P-gp inhibitor in the specific tissue are various, with different inhibiting P-gp activities [61,63,64]. In this view, WMP might significantly inhibit only P-gp mediated donepezil efflux through BBB, not in the intestine. In addition, it has been found that donepezil penetration through BBB were mediated by carrier-mediated transporters such as organic cation transporters (OCTs) and organic cation/carnitine transporter 2 (OCTN2), as well as passive diffusion within the brain [65,66]. Although further studies to investigate whether the effect of WMP on those carrier-mediated transporters of donepezil within the brain are necessary, the increase in OCT- and OCTN2-mediated donepezil uptake into the brain by WMP can be another possible mechanism for the increased donepezil concentration in the brain.

In conclusion, WMP increased donepezil concentration into the brain without a systemic exposure change in donepezil in mice. Additionally, there was little effect of WMP on donepezil distribution to tissues except in the brain. Considering the increased cell viability of Aβ_(25–35)_-induced neurotoxicity in SH-SY5Y neuroblastoma cells by DNP plus WMP treatment compared to DNP or WMP alone, our results revealed the potential of DNP plus WMP combination for AD treatment.

## 4. Materials and Methods

### 4.1. Chemicals and Reagents

Donepezil hydrochloride (DNP), finasteride [internal standard (IS) for liquid chromatography-tandem mass spectrometry (LC-MS/MS) analysis], and 3-(4,5-dimethylthiazol-2-yl)-2,5-diphenyltetrazolium bromide (MTT) were purchased from Sigma Aldrich (St. Louis, MO, USA). Aβ_(25–35)_ was purchased from Bachem Ltd. (St. Helens, UK). Minimum Essential Media (MEM), trypsin-EDTA were purchased from HyClone (Logan, UT, USA). Fetal Bovine Serum (FBS), antibiotic mixtures of penicillin and streptomycin were purchased from Life Technologies (Carlsbad, CA, USA). WMP was donated from the College of Pharmacy, Dongguk University (Seoul, Korea), and the UPLC-UV chromatogram of WMP was shown (Supplementary data 2). All other reagents were of analytical grade.

### 4.2. Preparation and Analysis of WMP

The water extract of pericarps of *G. mangostana* (WMP) was obtained from the sample prepared by the previous study [26]. The dried pericarp of *G.mangostana* L. (7.9 kg) was refluxed with water (7.9 L) for 2 h at 100 °C and WMP of 298.7 g was obtained.

The quantitative analysis using UHPLC-UV was operated by the following method. α-Mangostin, γ-mangostin, and the extract stock solution were prepared by dissolving the accurately weighed substances in methanol. The stock solutions of standards were diluted to appropriate concentrations in the ranges of 0.05–1.0 mg/mL (0.05, 0.1, 0.25, 0.5, 1 mg/mL) to obtain calibration curve.

Chromatographic separation of the analyte was performed on a Dionex Ultimate 3000 UHPLC system with DAD detector (Thermo Fisher Scientific, Waltham, MA, USA) and JH08S04-2545WT column (4.6 × 250 mm, S-4 μm, YMC, Kyoto, Japan). The wavelength was set 340 nm. The mobile phase consisted of 0.1% formic acid in water (A) and 0.1% formic acid in acetonitrile (B). The mobile phase consisting of (A) and (B) was delivered at a flow rate of 1.0 mL/min by the following programmed gradient elution: 67% (B, *v*/*v*) isocratic for 30 min, 67 → 80% (B) for 5 min, 80% (B, *v*/*v*) isocratic for 5 min, 80 → 100% (B) for 2 min, 100% (B) isocratic for 5 min, 100 → 67% (B) for 1 min, and 67% (B) isocratic for 2 min as post-run for reconditioning. The column temperature was maintained at 30 °C A 5μL aliquot was injected into the UHPLC system for analysis.

Calibration curve was established by plotting the peak areas versus concentrations of the analytes. Calibration curve for α-mangostin and γ-mangostin were = 64.795x + 870.14 (R^2^ = 0.9991) and y = 109.89x + 1008.6 (R^2^ = 0.9987). WMP was found to contain 0.95 % and 0.10 % (*w*/*w*) of α-mangostin and γ-mangostin, respectively, in WMP (Supplementary data 2).

### 4.3. Effect of WMP and DNP Combination on Cell Viability of SH-SY5Y Neuroblastoma Cells Treated with Aβ_(25–35)_

SH-SY5Y neuroblastoma cells were obtained from the Korean Cell Line Bank (Seoul National University of Hospital, Seoul, Republic of Korea). Cells were cultured in MEM supplemented with 10% heat-inactivated defined FBS, and an antibiotic mixture of 100 units/mL of penicillin and 100 μg/mL streptomycin, and grown at 37 °C in a humid atmosphere with 5% CO_2_.

To assess the effect of WMP on cell viability of SH-SY5Y neuroblastoma cells treated with Aβ_(25–35)_, MTT assay was conducted. To induce neurotoxicity by Aβ_(25–35)_ treatment, the cells were seeded (5 × 10^5^ cells/mL) in 96-well plates and incubated for 24 h at 37 °C. On the next day, cells were treated with Aβ_(25–35)_ (as a final concentration of 25 μM) before the treatment of DNP, WMP, or DNP plus WMP. A 100 μL of DNP, WMP, or DNP plus WMP in cell culture media was treated on the plates to achieve 10 μM of donepezil and 10 μg/mL of WMP as a final concentration, respectively, which was incubated for 24-h. Following the treatment, cell viability was assessed using MTT reduction assay. After adding 10 μL/well of MTT (5 mg/L) and incubating for 4 h, the supernatants were removed and 100 μL of DMSO was added in wells. After 1 h incubation at room temperature in the microshaker, the absorbance of the sample was measured by the microplate spectrophotometer reader (Molecular Devices, Sunnyvale, CA, USA) at 570 nm. The cell viability rate (%) was calculated as the absorbance of treated cells divided by that of control cells. The viability of the control cells was defined as 100%. Three independent experiments were performed in triplicate.

### 4.4. Animals

All animal studies were approved by the Institute of Laboratory Animal Resources of Dongguk University_Seoul (Approval number: IACUC-2016-004-1; Seoul, Korea) and conducted in accordance with the relevant guidelines. Male Institute of Cancer Research (ICR) mice (6 weeks old, approximately 30 g body weight) were purchased from the Orient Bio (Gyeonggi-do, Korea). Mice were randomly divided into three in each cage and housed under 12 h light/dark conditions with 20‒25 °C and 48–52% relative humidity. Diet and water ad libitum were provided. Mice were adaptively acclimated for two weeks before experiments.

### 4.5. Toxicity Test of WMP

WMP (dissolved in distilled water) at a dose of 20, 50, 100, or 500 mg/kg were orally administered to mice (*n* = 5 for each dose) once a day for seven days, respectively. Body weight and food/water consumption and behavioral change were observed every day.

### 4.6. Effect of WMP on the Pharmacokinetics of Donepezil

After 8 h fasting with free supply of water, mice were arbitrarily divided into DNP and DNPWMP groups. In the DNP group, donepezil hydrochloride (DNP) as a 10 mg/kg of donepezil (dissolved in distilled water) was administered by gavage to mice. In the DNPWMP group, the same dose of DNP (as 10 mg/kg of donepezil) and 300 mg/kg of WMP (dissolved in distilled water) were simultaneously administered by gavage to mice. The final concentrations of DNP and WMP were 2.19 mg/mL of DNP (containing 2 mg/mL of donepezil) and 60 mg/mL of WMP, respectively. Approximately 110 µL of blood sample was collected at each sampling time point, such as 0 (before the administration), 5, 15, 30, 60, 120, 240, and 360 min by cardiac puncture, and then centrifuged. A 50 µL of plasma was collected from the supernatant of each blood sample. Three or four blood samples were obtained per mouse, producing 10 sets of pharmacokinetic data. At 24 h, the metabolic cage was washed out by 5 mL of distilled water, and the fluid was combined into the urine collected over the previous 24 h. Each mouse was sacrificed by cervical dislocation, and then the entire gastrointestinal tract was removed, transferred into a beaker, and was cut into small pieces. After adding 10 mL of methanol, the content in the beaker was shaken and stirred for 1 min. A 1 mL aliquot for the supernatant was collected from each beaker and stored in –80 °C until analyzing samples by LC-MS/MS.

### 4.7. Effect of WMP on Tissue Distribution of Donepezil

DNP as 10 mg/kg of donepezil without and with a 300 mg/kg of WMP were orally administered by gavage to DNP and DNPWMP groups, respectively, the same method as the pharmacokinetic studies. The final concentrations of DNP and WMP were 2.19 mg/mL of DNP (containing 2 mg/mL of donepezil) and 60 mg/mL of WMP, respectively. At 1 or 4 h after their oral administrations, as much as blood was collected via the heart puncture and mice were sacrificed by loss of blood. After centrifugation, 50 µL of plasma was collected from each blood sample. Liver, stomach, small intestine, large intestine, kidney, and brain were excised, and approximately 1 g of each tissue (except 0.4 g of brain) was weighted. A 4-fold volume of normal saline was added to each tissue, which was homogenized and centrifuged at 12,000 rpm for 10 min. A 50 µL of the supernatant of each tissue was collected. All collected samples were stored at –80 °C until analyzing samples by LC-MS/MS. In addition, the tentative metabolites of donepezil in liver samples were conducted using LC-MS/MS.

### 4.8. Effect of WMP on Plasma Protein Binding of Donepezil

Protein binding of donepezil was measured using rapid equilibrium dialysis (RED) device (molecular weight cutoff of 8.0 KDa; Thermo scientific, Waltham, MA, USA). A 300 µL buffer solution, composed by 100 mM sodium phosphate with 150 mM sodium chloride, was inserted into one chamber, and 50 µL of plasma containing DNP or DNP plus WMP was inserted into another chamber in the RED device. DNP as 1 µg/mL of donepezil and 1 µg/mL WMP were the final concentration in the RED well plate, respectively. The RED device was incubated for 4 h at 37 °C and 250 rpm, and a 50 µL aliquot was collected from each chamber. Because donepezil was categorized as a positive drug with a high protein binding, a 1 µg/mL of metformin was additionally used as a positive compound as a low and plasma protein binding drug [44]. Experiment procedure for the protein binding of metformin as a positive drug were conducted as same as the case of donepezil. All samples were stored at –80 °C until analyzing samples by LC-MS/MS.

### 4.9. Effect of WMP on Donepezil Metabolism in In Vitro S9 Fractions of Various Tissues

The S9 fractions of various tissues were obtained, followed by the procedures in the previous report [67]. Liver and small intestine were excised and rinsed by normal saline. Each tissue was homogenized with 4-fold volume of 0.25 M sucrose, centrifuged at 9000 rpm and 4 °C for 20 min, and then the supernatant as S9 fraction of each tissue was obtained. The protein concentration of S9 fraction of each tissue was adjusted to 30 mg/mL. To measure the metabolic activity, a 135 μL of Tris-buffer (pH 7.4), 135 μL of S9 fraction of each tissue, 5 μL of 1 mM NADPH, and 5 μL of 3.3 mM UDPGA were added into the microtube, and the metabolic reaction was initiated by adding 2.5 μL of donepezil hydrochloride (50 μg/mL) with or without WMG (50 μg/mL). The final concentrations were DNP as 1 µg/mL of donepezil and 1 µg/mL WMP, respectively. This mixture in the microtube was incubated in a thermomixer at 37 °C and 500 rpm for 30 min. After a 30 min incubation, the enzyme metabolic reaction was terminated by adding a 100 μL of acetonitrile (including 250 ng/mL of the IS). After vortex and centrifugation of this sample at 12,000 rpm and 4 °C for 10 min, a 100 μL of the supernatant was obtained and the remaining donepezil concentration was determined by LC-MS/MS analytical method. In addition, the tentative metabolites of donepezil were identified using LC-MS/MS analytical method.

### 4.10. LC-MS/MS Analysis of Donepezil in Biological Samples

Donepezil concentrations in biological samples were monitored at Waters UPLC-XEVO TQ-S/UPLC system (Waters Corporation, Milford, MA, USA). The MS/MS operating system was positive mode with capillary voltages (3.0 kV), cone voltages (15 V), a source temperature of 650 °C, desolvation gas temperature of 350 °C, desolvation gas flow (650 L/h), cone gas flow (10 L/h), and collision gas flow (0.17 mL/min). The mass transitions for donepezil and IS were *m*/*z* 380.19 → 90.92 (collision energy, 40 eV) and 373.200 → 305.190 (collision energy, 30 eV) in the multiple reaction monitoring (MRM) mode with electrospray ionization (ESI) interface used to positive ions ([M + H])^+^. In the UPLC system, the mobile phase composition was started at 20:80 (*v*/*v*) of acetonitrile (A) and 0.1% formic acid in water (B) and gradually changed to 80:20 (*v*/*v*) for 4 min, and then switched back to 20:80 (*v*/*v*) for 6.5 min at a flow rate of 0.2 mL/min. The mobile phase composition was separated on the column (ACQUITY UPLC BEH C_18_, 2.1 mm × 100 mm i.d., 1.7 μm particle size; Waters, Dublin, Ireland) with a gradient mobile phase consisting of 0.1% formic acid in water and acetonitrile. A 50 μL of the biological sample was deproteinized by adding 100 μL acetonitrile containing 0.25 μg/mL of IS. After vortex-mixing and centrifugation, a 10 μL supernatant was injected into the column kept at 4 °C. All analytical data were processed using the Mass Lynx software (Version 4.1, Waters Corporation, Milford, MA, USA).

### 4.11. LC-MS/MS Analysis of the Tentative Metabolites of Donepezil

The metabolites of donepezil were tentatively identified in an in vitro metabolism and in vivo tissue distribution studies considering the different RTs, *m*/*z* ratios, and fragment ions identified by UPLC/MS and MS/MS. After conducting the sample preparation step of the in vitro metabolism study or in vivo tissue distribution samples, as mentioned above (Section 4.10), the tentative metabolites of donepezil were determined using the following analytical method.

The simultaneous full scan MS, MRM, parent scan, and daughter scan modes of a Waters UPLC-XEVO TQ system (Waters Corporation) were used to confirm the structures of any metabolites. The *m*/*z* ratios of the metabolites of donepezil were determined by full scans with positive modes ranging from *m*/*z* 500 to 900. Structural elucidation of the metabolites was based on the fragmentation patterns of the parent ions from the MS/MS mode at a collision energy of 20 eV, generating daughter ions. The mass transitions of donepezil were *m*/*z* 380.19 → 90.92 (40 eV for collision energy). The unknown masses were further analyzed in the MS/MS mode (i.e., daughter scan mode) with the ESI interface used to generate positive ions at capillary voltages (3.0 kV), cone voltages (15 V), a source temperature of 650 °C, desolvation gas temperature 350 °C, desolvation gas flow (650 L/h), cone gas flow (10 L/h), and collision gas flow (0.17 mL/min). The MRM methods included identification of possible metabolites generated by phase I and/or II reactions [43,67,68]. Based on the full scan and MRM modes, the parent *m*/*z* values of the possible metabolite candidates of donepezil were used to conduct daughter scan modes to identify the daughter ion fragments from the parent ion input. Daughter ion fragments were also used to confirm their parent ions using the scan mode.

The metabolites of donepezil and donepezil itself were separated on the column (ACQUITY UPLC BEH C_18_, 2.1 mm × 100 mm i.d., 1.7 μm particle size; Waters, Dublin, Ireland), with a flow rate of 0.2 mL/min. The mobile phase composition was separated with a gradient mobile phase consisting of 0.1% formic acid in water and acetonitrile: the mobile phase composition was started at 20:80 (*v*/*v*) of acetonitrile (A) and 0.1% formic acid in water (B) and gradually changed to 80:20 (*v*/*v*) for 13 min, switched back to the initial composition of 20:80 (*v*/*v*) for 13.1 min, and then maintained for 18 min. The total run time was 22 min. All analytical data were processed using the Mass Lynx software (Version 4.1, Waters Corporation, Milford, MA, USA).

### 4.12. Analysis of Pharmacokinetic Parameters

Standard methods [69] were used to calculate the pharmacokinetic parameters using a compartmental analysis with WinNonlin Professional software (version 2.1; Certara, Princeton, NJ, USA). As a PK model of donepezil after oral administration, a two-compartment model with first-order absorption and a linear elimination was used to establish a pharmacokinetic model of donepezil. The differential equations were as follows:

(1) $$\frac{dA}{dt}=-k_{a}\cdot A$$

(2) $$\frac{dX_{c}}{dt}=-k_{a}\cdot A-k_{12}\cdot X_{c}-k_{e}\cdot X_{c}+k_{21}\cdot X_{p}$$

(3) $$\frac{dX_{p}}{dt}=k_{12}\cdot X_{c}-k_{21}\cdot X_{p}$$

In the above equations, ka (1/min) is the apparent first-order absorption rate constant to the central compartment, and A (mg) is the drug amount in the absorption site. The *k*_12_ (1/min) and *k*_21_ (1/min) are apparent first-order intercompartmental distribution constants (or transfer rate constants), *k*_e_ (1/min) is the apparent first-order elimination rate constant from the central compartment, and *X*_c_ (mg) and *X*_p_ (mg) are the drug amount in the central and peripheral compartments, respectively. Peak plasma concentration (*C*_max_) and time to reach *C*_max_ (*T*_max_) were directly read from the data. All data are expressed as mean ± SD, except the median (ranges) used for *T*_max_.

### 4.13. Statistical Analysis

Student’s *t*-test was used to compare the two groups, and one-way ANOVA followed by Tukey’s post hoc test were used to compare multiple groups, respectively. Statistical analyses were performed with SigmaPlot 12.5 software (Systat Software, San Jose, CA, USA), and a *p* value of <0.05 was considered to be a significant difference.

## Figures and Tables

**Table 1:** Mean (± S.D.) pharmacokinetic parameters of donepezil after simultaneously oral administration of DNP (as 10 mg/kg of donepezil; *n* = 10 from 30 mice) without and with WMP (300 mg/kg; *n* = 10 from 28 mice) to mice, respectively.

Parameters	DNP (*n* = 10 from 30 Mice)	DNPWMP (*n* = 10 from 28 Mice )
Body weight (g)	28.30 ± 0.63	27.84 ± 0.54
AUC_360 min_ (μg min/mL)	16.21 ± 4.42	12.22 ± 2.43
AUC_0-∞_ (μg min/mL)	16.73 ± 4.16	12.60 ± 2.49
Terminal half-life (min)	77.32 ± 32.33	65.11 ± 10.83
C_max_ (μg/mL)	0.24 ± 0.12	0.16 ± 0.45
*T*_max_ (min)^a^	15 (15−15)	15 (15−30)
V_c_/*F* (mL/kg)	23,672.72 ± 7403.86	20,631.21 ± 1272.14
V_p_/*F* (mL/kg)	4160.44 ± 592.25	3886.46 ± 800.54
k_a_ (1/min)	0.071 ± 0.031	0.056 ± 0.028
k_12_ (1/min)	0.014 ± 0.010	0.012 ± 0.0042
k_21_ (1/min)	0.0087 ± 0.0058	0.010 ± 0.0062
k_e_ (1/min)	0.027 ± 0.013	0.023 ± 0.0043
*Ae*_0–24 h_ (% of dose)	1.65 ± 0.35	0.73 ± 0.67
*GI*_24 h_ (% of dose)	0.036 ± 0.02	0.08 ± 0.064

Three or four blood samples were obtained per mouse, which produced ten sets of pharmacokinetic data.

**Table 2:** Concentrations (μg/mL for plasma and μg/g for tissue) or T/P ratios of donepezil after oral administration of DNP (as 10 mg/kg of donepezil) with and without WMP (300 mg/kg) to mice (*n* = 5 for each group).

Tissue	1 h		4 h	
	DNP (*n* = 5)	DNPWMP (*n* = 5)	DNP (*n* = 5)	DNPWMP (*n* = 5)
Plasma	0.10 ± 0.05	0.10 ± 0.02	0.02 ± 0.01 *	0.01 ± 0.01 *
Brain	2.17 ± 1.21	1.98 ± 0.53	0.22 ± 0.08 *	0.36 ± 0.081 *^,+^
	(21.42 ± 6.07)	(20.44 ± 5.72)	(20.41 ± 5.72)	(31.73 ± 12.12) ^+^
Kidney	10.27 ± 6.55	7.91 ± 1.74	0.96 ± 0.17 *	0.90 ± 0.28 *
	(115 ± 64.79)	(82.37 ± 23.81)	(82.38 ± 23.79)	(78.27 ± 33.31)
Liver	15.07 ± 2.23	18.5 ± 2.34^+^	2.79 ± 0.70 *	3.53 ± 1.87 *
	(179 ± 85.28)	(191 ± 69.39)	(197 ± 69.42)	(285 ± 96.81)
Stomach	23.57 ± 5.79	35.48 ± 13.63	3.35 ± 1.53 *	4.75 ± 2.38 *
	(309 ± 196)	(379 ± 185)	(379 ± 185)	(426 ± 264)
Small intestine	15.82 ± 7.93	26.08 ± 9.60	3.33 ± 1.54 *	2.29 ± 0.97 *
	(177 ± 99.16)	(263 ± 84.92)	(263 ± 84.88)	(189 ± 63.79)
Large intestine	15.33 ± 5.50	22.41 ± 13.92	2.39 ± 0.62 *	1.58 ± 0.40 *^,+^
	(183 ± 100)	(218 ± 119)	(218 ± 119)	(147 ± 73.40)

Values in parentheses are mean ± S.D. values of the tissue to plasma (T/P) ratios; * statistically different (*p* < 0.05) between 1 and 4 h in same treatment group; + statistically different (*p* < 0.05) between DNP and DNPWMP groups at same time.

## Data Availability

The data presented in this study are available on request from the corresponding author. The data are not publicly available due to conducting the further investigation based on the data.

## Supplementary Materials

The following are available online, Supplementary data 1. Fragment ion spectra of the tentative metabolites (M1–M7) and donepezil by product ion scan mode. (A) donepezil, (B) M1, (C) M2 and M3, (D) M4 and M5, and (E) M6 and M7; Supplementary data 2. UHPLC-UV chromatogram of γ-mangostin and α-mangostin in WMP. As a quantitative analytical result, WMP was found to contain 0.95 % and 0.10 % (w/w) of α-mangostin and γ-mangostin, respectively, in WMP.
