# Supplementary material for: Effect of Water Extract of Mangosteen Pericarp on Donepezil Pharmacokinetics in Mice
Source: Molecules. 2021 Aug 30;26(17):5246. doi: 10.3390/molecules26175246 (PMC8434012; doi:10.3390/molecules26175246)

Supplementary data 1. Fragment ion spectra of the tentative metabolites (M1-M7) and donepezil by product ion scan mode. (A) donepezil, (B) M1, (C) M2 and M3, (D) M4 and M5, and (E) M6 and M7.

(A) donepezil: Product ion scan for *m/z* 380

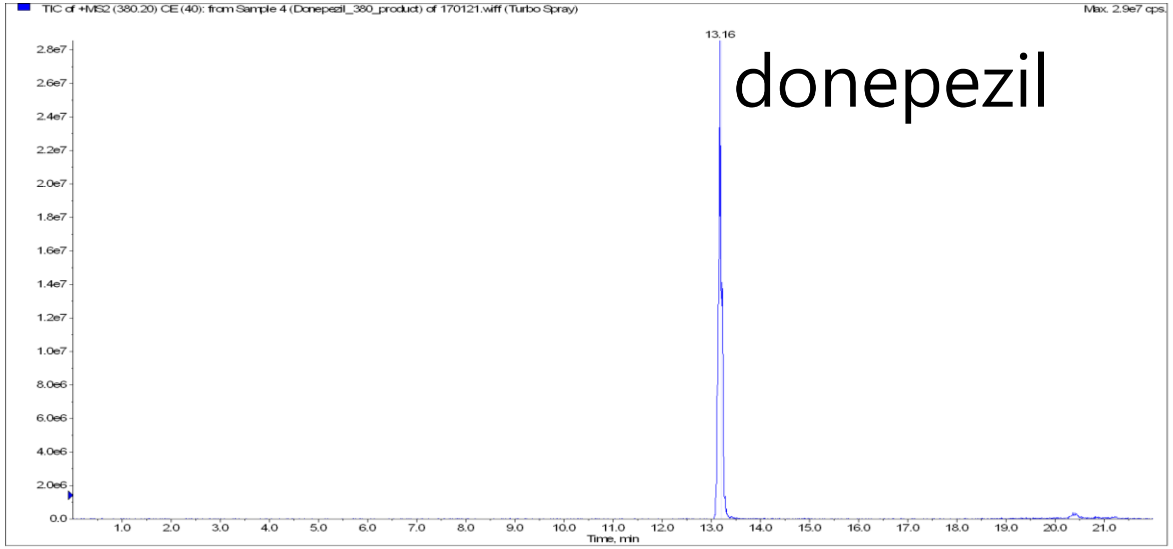

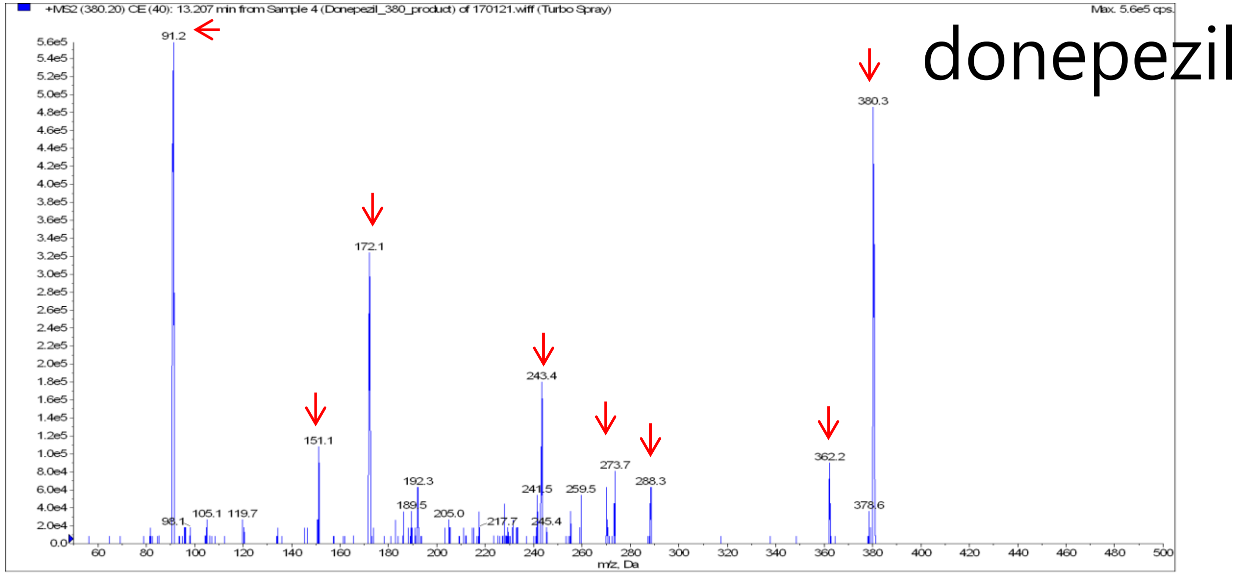

(B) M1: Product ion scan for *m/z* 542

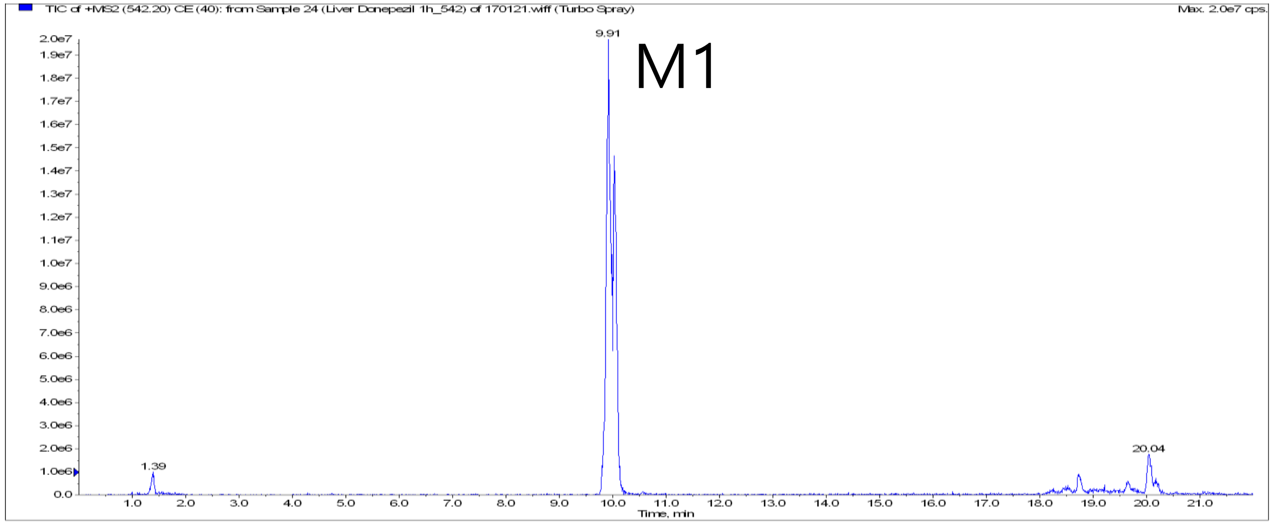

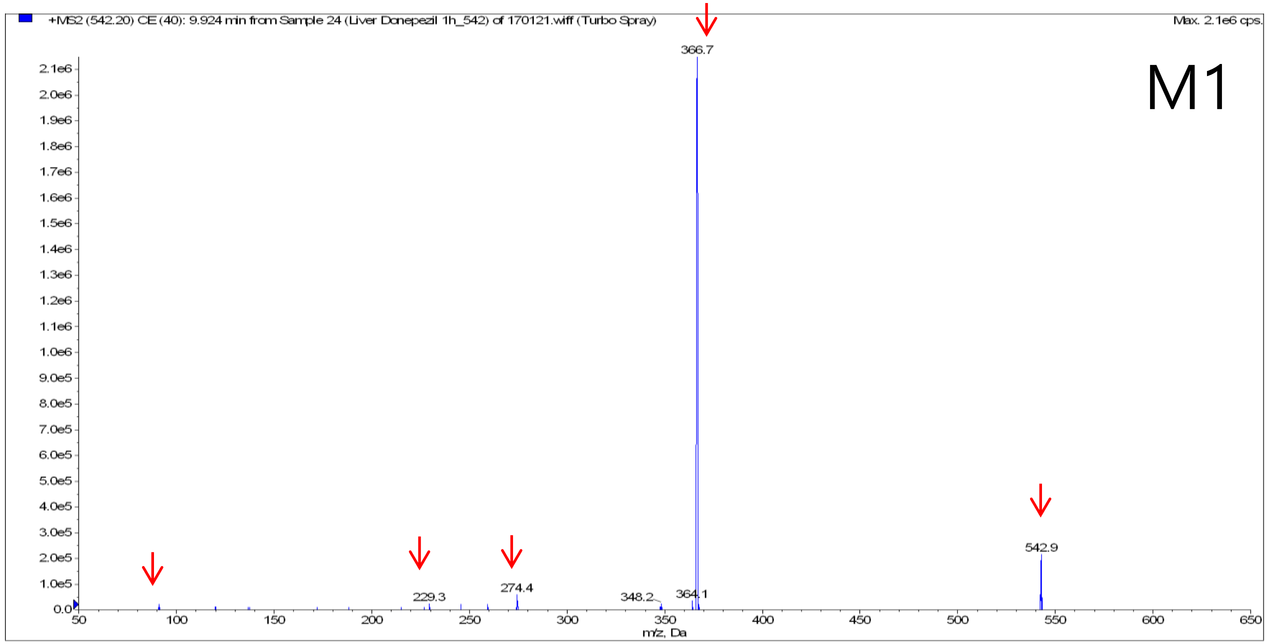

(C)M2 and M3: Product ion scan for *m/z* 366

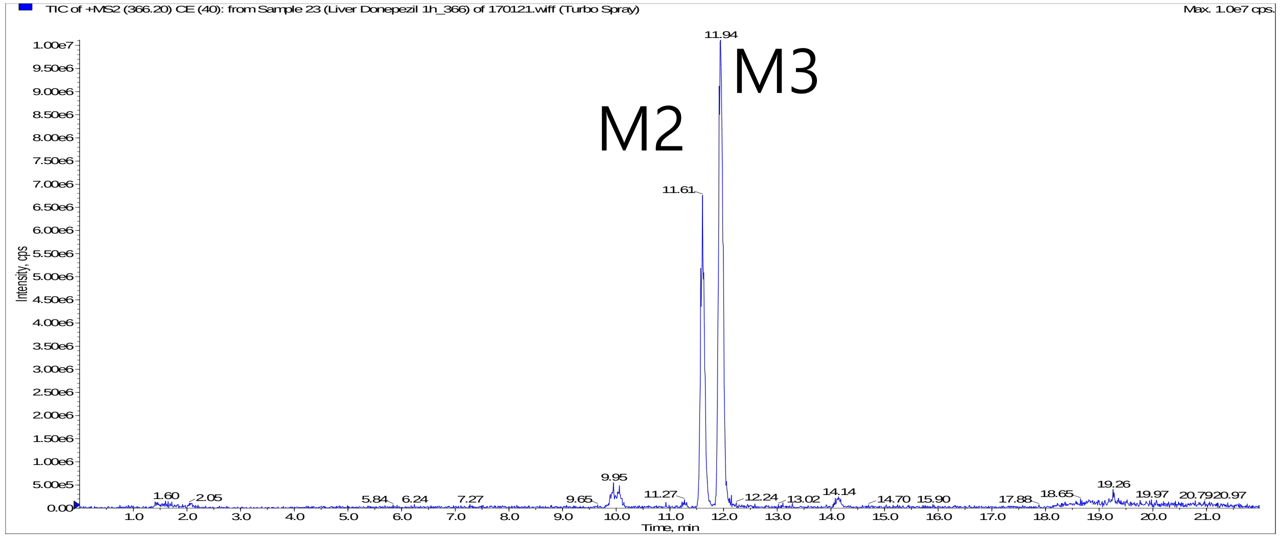

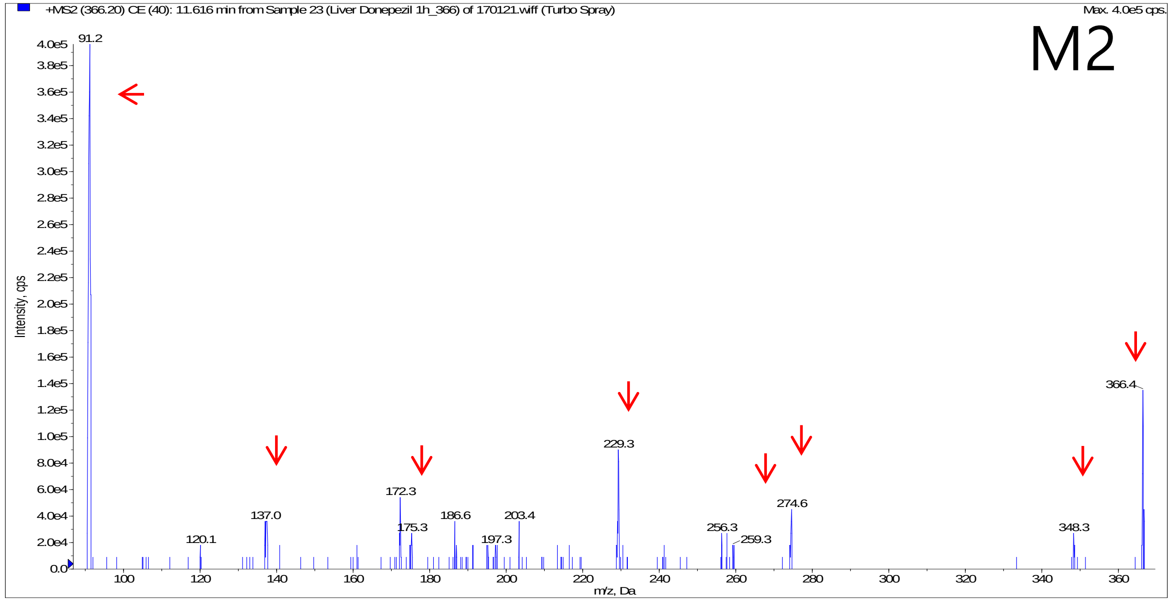

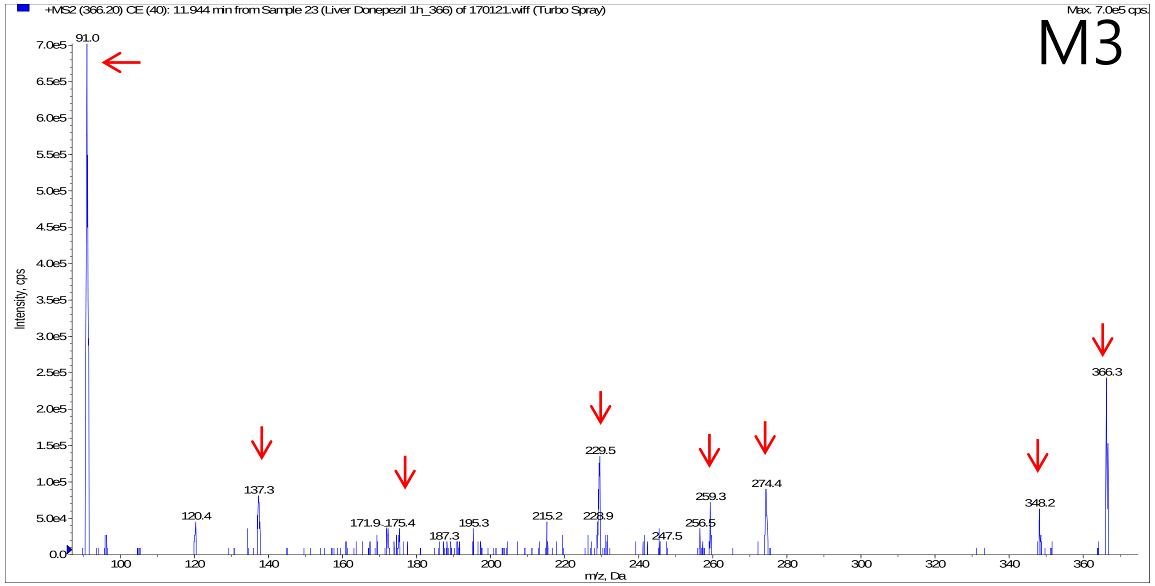

(D) M4 and M5: Product ion scan for *m/z* 396

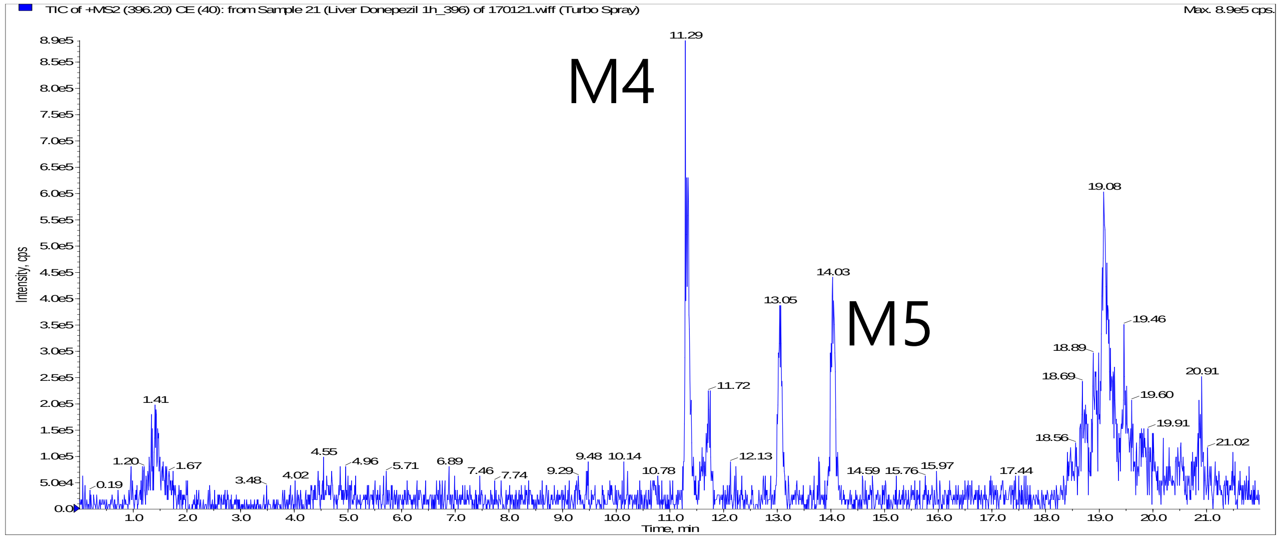

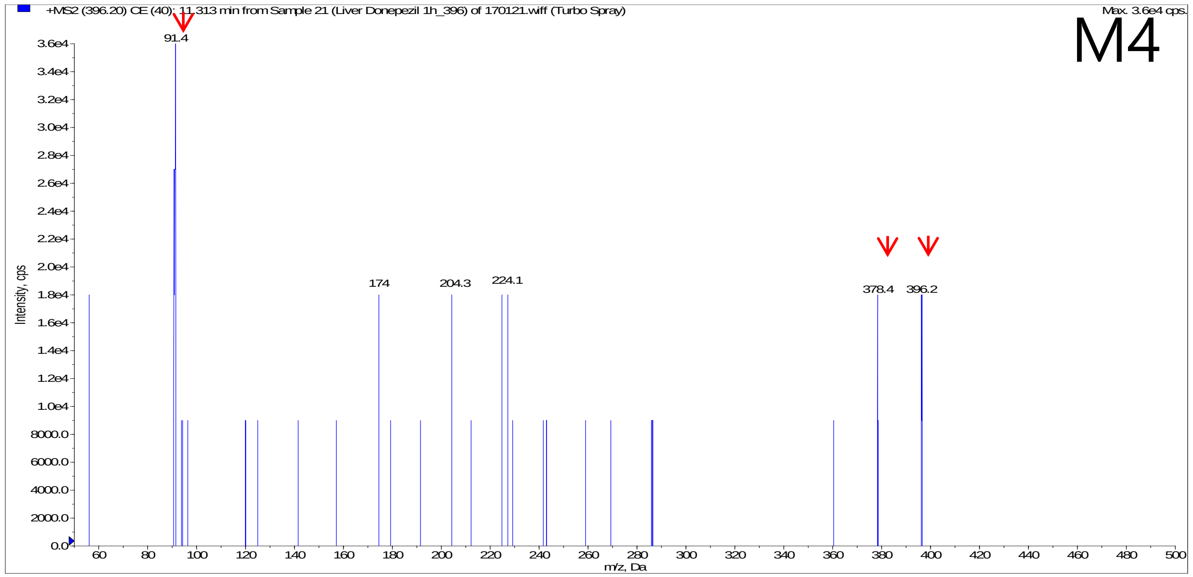

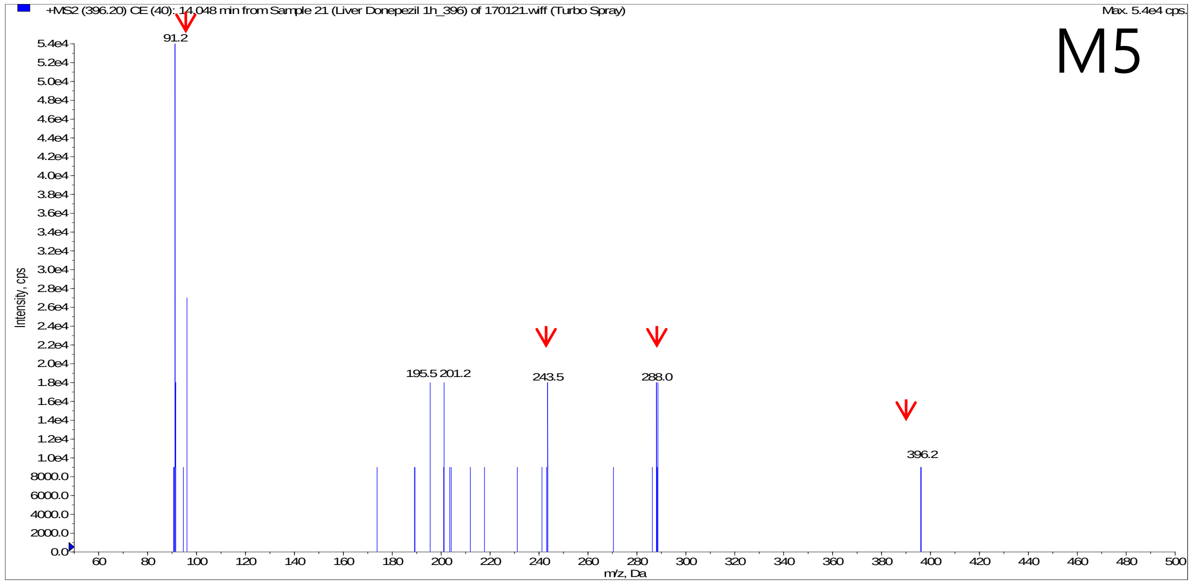

(E) M6 and M7: Product ion scan for *m/z* 396

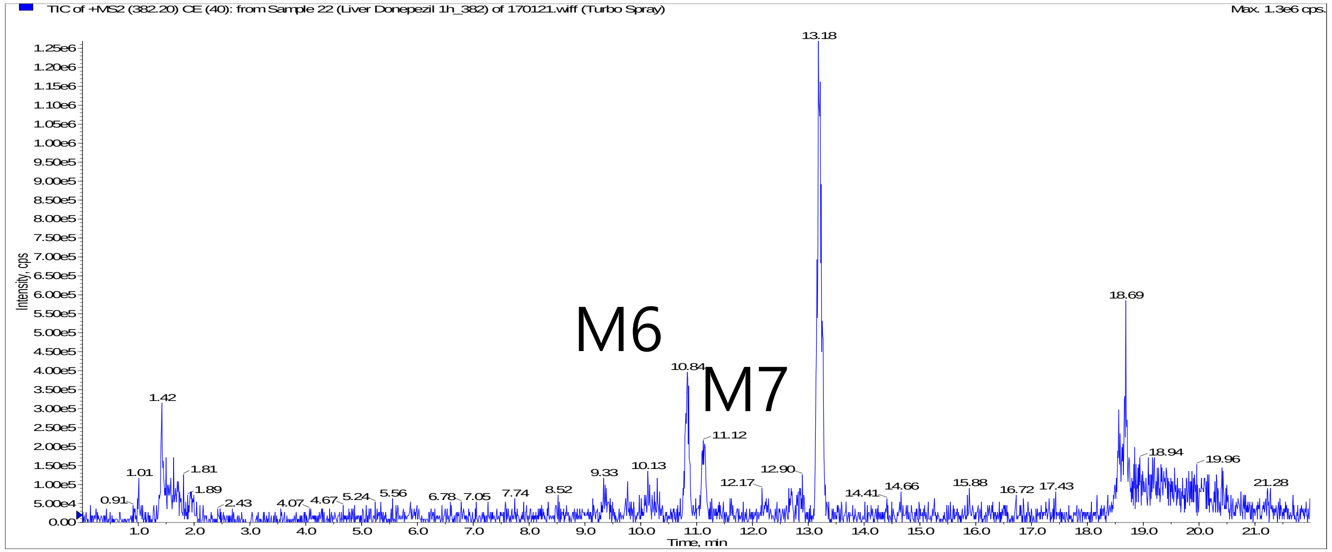

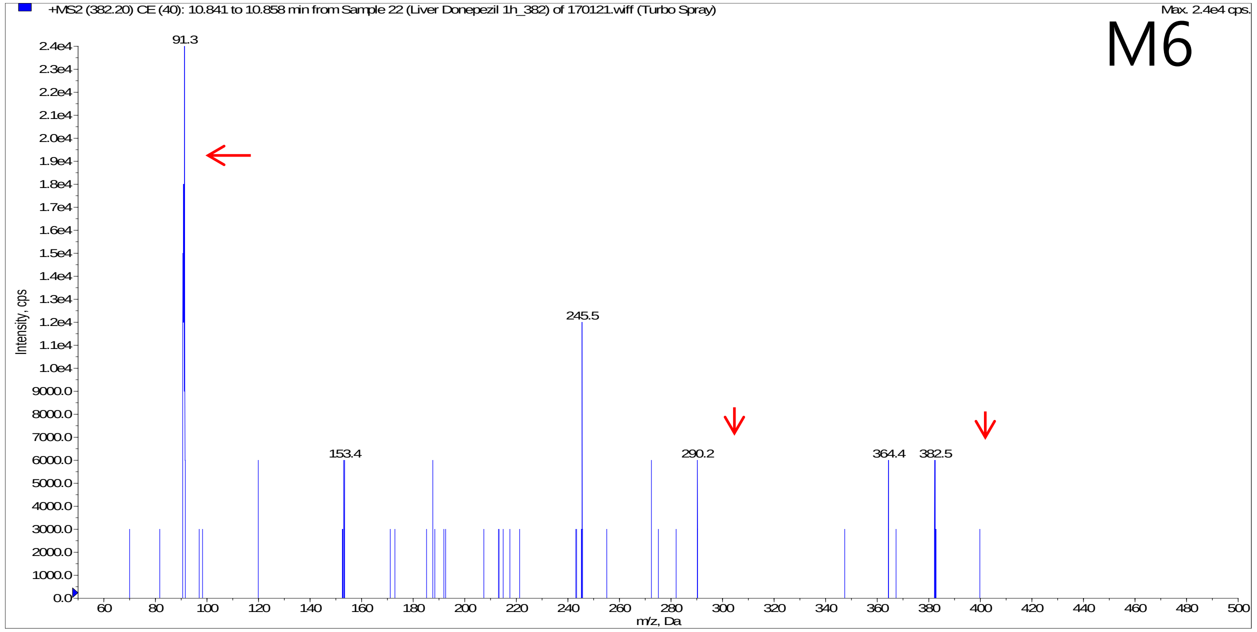

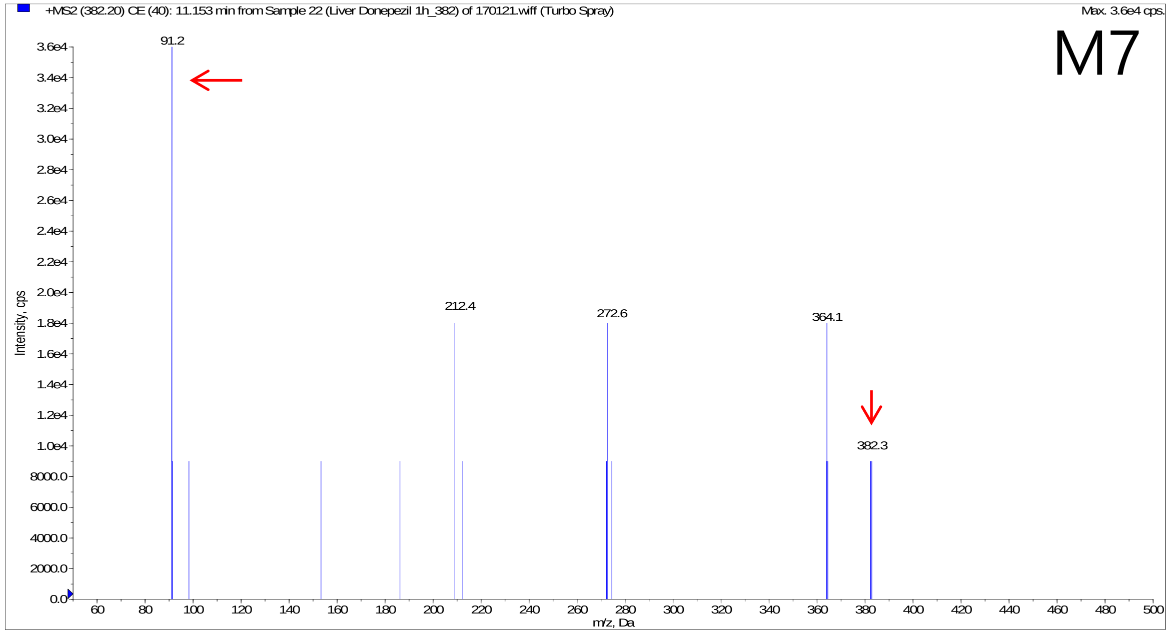

Supplementary data 2. UHPLC-UV chromatogram of γ-mangostin and α-mangostin in WMP. As a quantitative analytical result, WMP was found to contain 0.95 % and 0.10 % (w/w) of α-mangostin and γ-mangostin, respectively, in WMP.

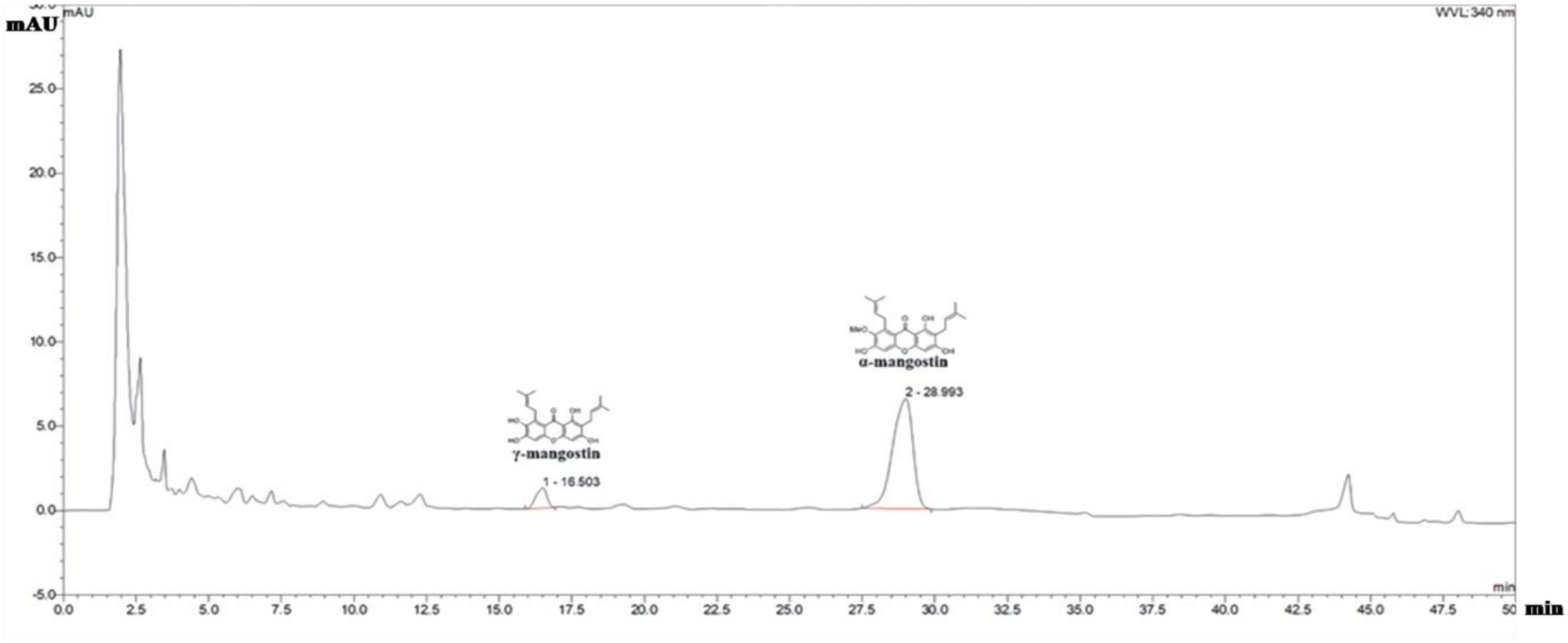

Supplement: Supplementary file 1 [file molecules-26-05246-s001.zip › molecules-1335986-supplementary.pdf]
